# How the COVID-19 Pandemic Alters the Landscapes of the HIV and Tuberculosis Epidemics in South Africa: A Case Study and Future Directions

**DOI:** 10.3390/epidemiologia3020023

**Published:** 2022-06-06

**Authors:** Daniel Eike, Maximilia Hogrebe, Dagem Kifle, Miriam Tregilgas, Anshu Uppal, Alexandra Calmy

**Affiliations:** 1 Global Studies Institute, University of Geneva, 1205 Geneva, Switzerland; maxmilia.hogrebe@etu.unige.ch (M.H.); dagem.kifle@etu.unige.ch (D.K.); miriam.tregilgas@etu.unige.ch (M.T.); anshu.uppal@etu.unige.ch (A.U.); 2 HIV/AIDS Unit, Division of Infectious Disease, Geneva University Hospitals, 1205 Geneva, Switzerland; alexandra.calmy@hcuge.ch

**Keywords:** COVID-19, South Africa, HIV, tuberculosis, inequality, health disparity, health access

## Abstract

South Africa has long grappled with one of the highest HIV and tuberculosis (TB) burdens in the world. The COVID-19 pandemic poses challenges to the country’s already strained health system. Measures to contain COVID-19 virus may have further hampered the containment of HIV and TB in the country and further widened the socioeconomic gap. South Africa’s handling of the pandemic has led to disruptions to HIV/TB testing and treatment. It has, furthermore, influenced social risk factors associated with increased transmission of these diseases. Individuals living with HIV and/or TB also face higher risk of developing severe COVID-19 disease. In this case study, we contextualize the HIV/TB landscape in South Africa and analyze the direct and indirect impact of the COVID-19 pandemic on the country’s efforts to combat these ongoing epidemics.

## 1. Introduction

South Africa’s healthcare system was deeply burdened by the onslaught of the SARS-CoV-2 virus (COVID-19) [1]. The country experienced a harsh lockdown in early 2020 [2]. The burden of the lockdown was greatest in the poorest areas of South Africa where promiscuity and lack of proper sanitation became major problems [1]. Exiting the lockdown, the country was tasked with containing the deadly virus while concurrently battling the ongoing human immunodeficiency virus (HIV) and tuberculosis (TB) epidemics that have burdened the country’s healthcare system for decades.

HIV and TB have had the highest global mortality rates of all infectious diseases since 2018 [3]. The burden of these diseases disproportionately affects low- and middle-income countries (LMIC), notably sub-Saharan Africa. Health professionals in LMICs report that more than 40% of HIV and TB patients experienced hindered or fully restricted access to health facilities throughout the COVID-19 pandemic [3].

South Africa has the highest number of people living with HIV in the world; approximately 20% of the adult population (15–49 years) is HIV positive [4,5]. Furthermore, there are an estimated 320,000 people suffering from TB and close to 80,000 TB deaths annually [4]. Coinfection of HIV and TB worsens disease outcomes and increases overall disease burden. A quarter of South Africa’s national health budget is allocated to HIV, maternal health, and TB [4].

This paper will present the implications of the COVID-19 pandemic on the ongoing HIV and TB epidemics that have ravaged South Africa over the past few decades. The government and populace of South Africa have a long history of fighting infectious diseases. With the introduction of COVID-19 to South Africa in early 2020, the country faced a host of novel problems. The pandemic hampered efforts to control the pre-existing HIV and TB epidemics. HIV- and TB-positive individuals face greater risk for developing severe COVID-19 infection [6]. Government lockdowns, social stigmas, and distrust in healthcare services interfere with access to quality care. A major fear during this pandemic has been that individuals with HIV/TB may not only face a greater risk of developing severe COVID-19 infection, but also face severe consequences from disruptions to testing and treatment services. For example, among HIV patients already receiving antiretroviral therapy (ART) in sub-Saharan Africa, a 6-month disruption of ART drugs across 50% of individuals was predicted to lead to a 63% increase in HIV-related deaths over a 1-year period, as well as a 60% increase in mother-to-child transmission of HIV [7].

The following sections will investigate South Africa’s existing healthcare system, history of HIV and TB epidemics at the country level, and the handling of COVID-19 from its beginnings in March 2020 until the present. The study will then investigate the impact and influence of COVID-19 on health policy, health systems, and individuals in the context of ongoing HIV and TB epidemics which are ever-present in South African society.

## 2. Materials and Methods

This case study uses three major types of information source to perform its review: scientific literature, country reports, and news articles. Scientific experiments, reviews, and conference abstracts have been searched through well-established engines, with a majority coming from PubMed, ScienceDirect, Google Scholar, and MedRxiv. News articles and country reports, whether produced nationally or through other agencies (private, governmental, intergovernmental, or non-profit), were collected primarily online. The credibility of information providers and times of publication were important selection criteria for country reports and news articles to ensure the validity and relevance of data. The SMART method of evaluating sources was used to assess the credibility of sources to verify content creators, motives behind what is written, the authority responsible for such information, coherence in the information reviewed, and alignment with other news sources. Examples of sources used for the data and statistics include Statistics South Africa (Government of South Africa) and the World Bank.

Keywords were entered into the selected search engines. As this work primarily investigates the impact of COVID-19 on HIV and TB within South Africa, the following primary keywords were entered: COVID-19, South Africa, HIV, tuberculosis, inequality, health disparity, and health access. The number and order of words were varied, as were the diseases, which were entered either by full-name or as an acronym. Other search terms related to the primary keywords were searched, for example, in relation to COVID-19 vaccines, history of South Africa, and stigmas associated with HIV and TB. We were careful to select only academic literature that directly addressed the research question. All sources of information selected for this report were in English.

The topics discussed in this work are dynamic and changing through various economic, social, and political mechanisms (for example, the vaccination campaign, health budget for South Africa, etc.). For this reason, it is important to mention that this review is based on the situation observed in South Africa as of the end of 2021.

## 3. Results

### 3.1. Case Presentation

#### 3.1.1. Demographic, Economic, Geographic, and Political Characteristics of the Country

South Africa is a constitutional multiparty democracy with a three-tier system of government, meaning the government is divided into three levels, the national, provincial, and local governments [8,9]. It has a population of 60.14 million, of which, 81% identify as black African, around 8% as white, approximately 9% as colored, and the remainder as Indian/Asian. Around three-fifths of the population is under the age of 30. Due to its ethnic diversity, there are eleven official languages in South Africa and a multitude of different cultures [8,9]. South Africa covers an area of 1,220,813 km^2^. Gauteng is the smallest province with an area of 18,178 km^2^ but is home to the largest share of the population, with a population density of 852 people per square kilometer [9]. Furthermore, South Africa still has a high number of informal settlements, with high population densities, high poverty rates, and often insufficient access to water and sanitation [10]. According to Minister Kubayi, this movement of urbanization resulting in informal settlements is further aggravated by large numbers of non-South African migrants who move to South African cities in search of improved economic opportunities [11] 6/2/2022 4:52:00 PM. As a result, many South African cities suffer from inadequate infrastructure to accommodate the ever-growing urban population.

The legacy of apartheid (government mandated separation of race) is still ever-present in South Africa. It has instilled widespread inequalities along racial lines in the South African population [12,13]. White South Africans benefitted from a significantly better education system throughout apartheid [13,14]. The same principles applied to the health system and many other aspects of life [13]. Today, in post-apartheid South Africa, education inequalities still largely follow racial lines [14]. South Africa consistently has one of the highest income inequalities in the world [15]. The most recent Gini coefficient recorded by the World Bank for South Africa in 2014, was 63.0 [16]. Zero represents perfect income equality and 100 represents complete income inequality.

High levels of poverty and unemployment have left their mark on the South African population and economy [17], whereby, poverty follows ethnic boundaries and mainly affects black Africans and people of color. Young people suffer from especially high rates of poverty. South Africa’s recorded unemployment rates reached an all-time high of 34.4% for the second quarter of 2021 [18].

South Africa has experienced low economic growth for several years and its economy took a major downturn during the COVID-19 pandemic, as the GDP contracted by 6.96% in 2020 [15,19]. The severe impact on the economy is expected to further exacerbate poverty in South Africa.

#### 3.1.2. Overview of South Africa’s Healthcare System

Prior to the COVID-19 pandemic, South Africa’s health system was already strained by decades of coping with a quadruple burden of disease: (i) co-incidence of HIV/AIDS and TB, (ii) a rise in obesity and non-communicable diseases, (iii) continuing poor outcomes for child, infant, and maternal health, and (iv) injury and violence [20]. In keeping with the enduring inequalities linked to the legacy of apartheid and colonial rule, much of this burden has long been disproportionately borne by poor, black households [20].

With the end of apartheid in 1994, the new government quickly sought to address deeply rooted inequalities in the context of health; the 1996 constitution explicitly guaranteed the rights of all South Africans to have access to healthcare services, food and water, and social security, and importantly, mandated that “no one may be refused emergency medical treatment” [21]. This provision put a strong onus on the state to actively take measures to realize each of these rights, and in the early years of the new democracy, the government made great strides to fundamentally reform the healthcare system and promote equitable health access. These initiatives included consolidation of health administrations into a single national department, the introduction of a district-based primary health system, a major infrastructure plan to upgrade and build new clinics, free health care to pregnant women and children under the age of 6 years, and free primary care for all [20,22]. Despite these efforts over the last couple of decades, there are marked differences in health access and outcomes along socioeconomic strata, resulting from persistent systemic and structural challenges.

South Africa continues to operate a highly disparate two-tier health system, consisting of a large and poorly resourced public sector that serves the majority of its population, and a small, well-resourced private sector that caters to a wealthy demographic (those who can pay for services out-of-pocket and 17% who are covered by medical aid schemes) [23]. Despite serving over 80% of the population, the public sector accounts for less than half of national health expenditure [22] and is plagued by a perennial shortage of health workers, especially in rural and underserved areas [24]. South Africa has one of the highest concentrations of health workers on the continent, but public sector workers are often overburdened and face poor working conditions, leading many to seek opportunities abroad or in the private sector [24,25]. For example, of the total registered medical practitioners in the country, only about 30% work in the public sector [22], and this exacerbates the socioeconomic disparities in health access [26].

In contrast, the private sector is capable of providing a standard of care that is comparable with the health systems of highly developed countries [26]. Access to the private system is drastically split by race and socio-economic status. Until the 1970s, membership to private medical aid schemes was restricted to white people [20]. This restriction has left a lasting impact: only 17% of people were covered by medical aid schemes in 2019, but even within this small number the coverage rates differ widely between the population groups, with black Africans having the lowest (10.8%), followed by colored individuals (20.6%), Indian/Asian individuals (47.2%), and white individuals having the highest (72.4%) [23].

#### 3.1.3. Historical Context of HIV and Tuberculosis in South Africa

Since the first case of HIV in 1982, South Africa’s healthcare system has been severely overburdened by the HIV epidemic. The government failed to launch a sufficient national response to the growing epidemic, as it was hindered by the anti-apartheid regime under Thabo Mbeki (1999–2008) which negated the threat of the epidemic by promoting it as a disease common among homosexuals [27]. Under Mbeki’s presidency, supported by then Health Minister Manto Tshabalala-Msimang, the government maintained a detrimental attitude to ART, disseminating information indicating that ART and poverty were direct causes of HIV [28]. These health policy decisions are reflected in HIV incidence rates over the past decades: in 1990, South Africa experienced a dramatic increase in HIV prevalence from 0.76% 10.44%, then to 22.4% by 1995, finally resulting in 40% of all adult deaths in 2000 [27]. In 2000, access to and distribution of ART drugs to treat HIV finally increased due to the production of generic versions, resulting in a dramatic reduction in price [29].

**Figure 1:**
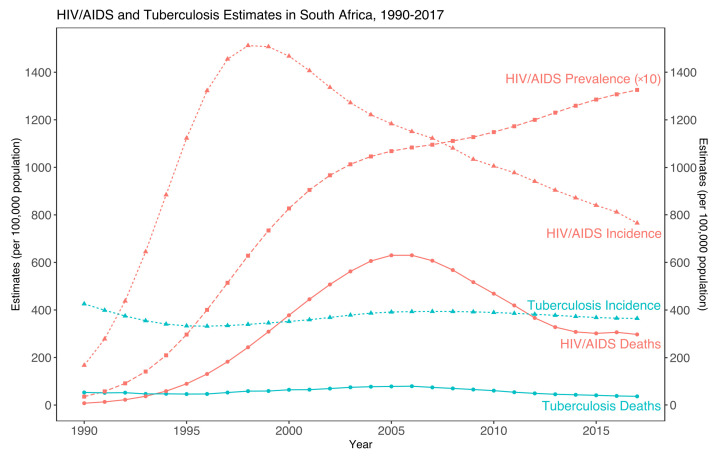
Historical overview of the HIV/AIDS and TB epidemics in South Africa, 1990–2017. Prevalence of HIV/AIDS is divided by 10 for scale. (Adapted from source: [31]).

Regarding TB, the Directly Observed Treatment Strategy (DOTS program) was introduced by the WHO in 1994, and adopted by South Africa to control and fight the TB epidemic. However, the HIV epidemic has resulted in a sharp increase in TB rates from the late 1990s: TB cases have increased from 163 cases per 100,000 in 1986 to 628 cases per 100,000 in 2006 (Figure 1) [30].

Today, multiple national government and international programs are in force to combat HIV in South Africa. Most important to mention are the UNAIDS 90-90-90 targets, the national HIV testing and counselling (HTC) campaign, and the ART program. The UNAIDS 90-90-90 goals aim to make 90% of those infected with HIV aware of their status, 90% of those to receive treatment, and 90% of those to be virally suppressed. The first target has been reached, but treatment and suppression goals have not been fulfilled. The late implementation of the ART programs and the Prevention of Mother-to-Child Transmission program (not until 2002) is traced to governmental denial, resulting in a lost opportunity of reaching 50% of HIV infected people on ART [28]. This is still evident in South Africa’s HIV/TB burden today. However, by 2004, ART had become more widespread. It is now the largest HIV treatment program in the world, providing treatment to 4.5 million people [5,30].

South Africa still faces an uphill battle to control the co-occurring HIV and TB epidemics. With approximately 7.8 million people infected with HIV, and a prevalence of 20% among adults, South Africa has one of the largest concentrations of people living with HIV in the world [32]. In 2017, 28,700 cases of TB resulted in death, making TB the deadliest communicable disease in South Africa [33]. The high rate of HIV cases, of which approximately 56% are TB/HIV co-infections, represent a severe double burden in South Africa [5].

South Africa’s National Strategic Plan (NSP) for HIV, TB, and sexually transmitted infections (STI) 2017–2022, is a strategic framework to further reduce the morbidity and mortality in the main populations associated with HIV, TB and STIs in South Africa. The NSP targets nine goals, amongst them, to reach vulnerable populations with customized interventions (Goal 3), as well promoting leadership and shared accountability for a sustainable response to HIV, TB, and STIs (Goal 6) [34]. South Africa has achieved successes with the 2012–2016 NSP, including: sexual transmission of HIV among 15–49 year-olds decreasing by 34% from 410,000 per year in 2011 to an estimated 270,000; HIV transmission from mother to newborn decreased from 3.5% in 2010 to 1.8% in 2014; and TB treatment success rate rose to 83% in 2016 [34]. Nevertheless, it can be observed that the incidence of TB has not consistently decreased since 2010. The number of multidrug-resistant TB (MDR-TB) cases has doubled from 7350 cases in 2007 to 14,161 in 2012, representing a heavy burden, also in the face of future developments in HIV/TB treatment [35].

HIV prevalence in South Africa is unevenly distributed, and communities such as men who have sex with men (MSM), transgender individuals, and sex workers experience extensive stigma and discrimination. Access to and quality of health care is severely limited in these communities [36]. The national average HIV prevalence among sex workers is 57.7% and among MSM is 18.1%. However, these figures are dependent on geographic differences and socioeconomic status [37]. Social stigmas around HIV are fueled by traditional, conservative attitudes among the elite, leading to widespread discrimination based on race, sexual orientation, and gender. HIV prevalence is significantly higher among LGBTQI+ communities and female sex workers; these communities, therefore, experience an increased risk of HIV-related violence and hate crimes [36,38]. Stigmatization of TB/HIV co-infected persons include poverty, drug abuse, homosexuality, and sex work [39]. The term “the paradox of dual illness” describes the strong association of HIV infections with TB in Africa [40]. Daftary claims, “the greater negative social desirability, labelling and stigma reserved for people with HIV were transferred to individuals with TB. The identity associated with TB became as undesirable and stigmatized as with HIV” [40]. A wide gender gap in HIV prevalence should be noted, with an incidence of 26% of women as opposed to 15% of men in 2017. Poverty, low status of women, and gender-based violence contribute to this disparity [37]. However, the gender disparity of HIV is the opposite: men have a 25% higher risk of death from HIV than their female counterparts [41].

#### 3.1.4. Impact of COVID-19 on Country Level

In early 2020, the SARS-CoV-2 virus/COVID-19 was detected in Wuhan, China and promptly swept across much of the world. It was declared a pandemic by the World Health Organization (WHO) on 11 March 2020 [42]. South Africa has absorbed the brunt of the COVID-19 pandemic on the African continent. It accounts for 34.1% (2.92 million) of total reported COVID-19 cases and 40.6% (89,387) of deaths related to COVID-19 in Africa [43]. However, official numbers from Africa are criticized for their unreliability due to under-reporting and lack of testing infrastructure. When standardized with global age-specific fatality rates and adjusting for Africa’s age demographic, death rates on the continent could be four times that of reported figures [44]. In South Africa specifically, where the median age is higher (27 versus 18 years in sub-Saharan Africa), and testing infrastructure is more robust, it is still estimated that excess deaths could be three times higher than officially reported figures [45].

The first case of COVID-19 in South Africa was reported by the (South African) National Institute for Communicable Diseases on 5 March 2020 [46]. A South African man tested positive upon returning from a trip to Italy [46]. By 15 March 2020, President Cyril Ramaphosa declared a national state of disaster. Immediate travel restrictions and school closures were announced.

A swift increase in cases prompted the government to announce a strict national lockdown effective from 26 March to 16 April 2020 [1]. This was later extended to 30 April 2020. Upon announcement, the total number of cases in the country had risen sharply to 554 [1]. No deaths had been reported at this point. The initial lockdown in South Africa was the strictest on the African continent. People were only allowed to leave their homes for medical reasons and essential grocery shopping. All social gatherings, sports, and outdoor activities were banned. The provisions of the lockdown also included a total ban on the sale of cigarettes and alcohol [1]. President Ramaphosa unveiled a plan to gradually “reopen” South Africa, beginning on 1 May 2020, via a five-level “alert system” determined by several criteria, including: transmission rate, infection level, health facility capacity, and economic/social impacts of restrictions [47]. A summary of the five alert levels is as follows: 5—strict lockdown to contain the spread of the virus, only essential travel allowed outside of homes; 4—some activity allowed, subject to extreme precautions to limit community transmission; 3—easing of restrictions on work and social activities, but caution maintained over a high risk of transmission; 2—further easing of restrictions but maintenance of physical distancing measures; and 1—most normal activities could resume, with precautions and guidelines followed at all times [47]. Over the next several months, the country oscillated between levels of restrictions according to these criteria but did not return to its initial strict lockdown measures (Level 5).

The (Level 5) lockdown was initially met with widespread praise by South Africans, especially among the middle and upper class [1]. Government approval ratings increased during this period [48]. A contrasting rhetoric emerged in poorer areas, where the lack of medical resources, food shortages, and financial resources led to widespread looting, hunger strikes, and clashes with local police [1]. As the pandemic wore on, government approval dropped, influenced by reports of corruption and financial mismanagement of relief funds [48].

**Figure 2:**
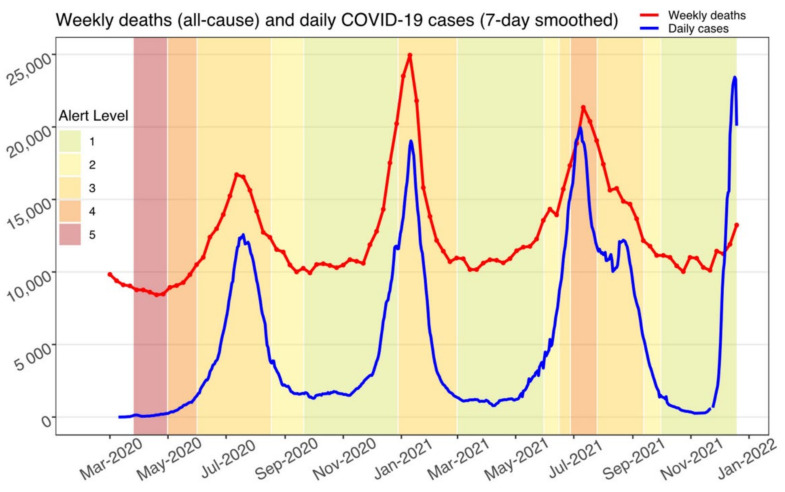
Weekly deaths (all-cause) and new confirmed COVID-19 cases, with shading to indicate the different “alert level” periods in South Africa. (Adapted from sources: [47,49,50,51]).

The timeline of the COVID-19 pandemic in South Africa is characterized by three distinct waves of infection (Figure 2). The first wave occurred from the end of June–August 2020, the second from December 2020–February 2021, and the third from May–October 2021. A peak in daily new cases was reached on 8 July 2021 with a total of 19,956 new cases reported.

Widespread riots in early July 2021, catalyzed by the jailing of former president, Jacob Zuma, are thought to have exacerbated the third wave of infection [52]. Widespread arson, looting, and roadblocks plagued the country, forcing health and testing centers to shut. Patients and health staff were unable to reach healthcare facilities during this period [52].

As of 10 November 2021, there had been 2,924,317 infections and 89,387 deaths from COVID-19 reported in South Africa [43]. New infections and deaths are currently 1% of the 8 July 2021 peak and falling [53].

South Africa’s vaccination campaign began on 17 February 2021, with a goal of vaccinating 67% of the population by the end of 2021 [54]. The campaign was swiftly halted over concerns that the received AstraZeneca vaccine was ineffective against the beta variant which originated in the country in October 2020 [55]. Continued roll out of vaccines has been slow and the country remains firmly behind its 67% goal with only 19.9% (23,264,992 doses) of the population vaccinated as of 10 November 2021 [53].

The COVID-19 pandemic disproportionately impacted underserved communities in South Africa. Data collected in Cape Town demonstrate that low-income sub-districts suffered a higher age-standardized death rate than middle- and high-income sub-districts [56]. Income-related health inequality was estimated to be six times higher than in 2017 [57]. Class inequality overburdens those living in townships across South Africa; people living in these areas are often unable to social distance and sanitation is generally poor [58]. Furthermore, access to adequate healthcare for this population is limited as many are limited to the overburdened public health system [58].

### 3.2. Current Impact and Long-Term Influence of the COVID-19 Pandemic on Global and Public Health, Public Policy, and Healthcare Systems

#### 3.2.1. The Indirect Consequences of the COVID-19 Measures

The impacts of self-isolation on health during COVID-19 lockdowns have been extensively discussed. Among the most reported issues of self-isolation is an increase in cases of domestic abuse, particularly against women. South Africa is already known to have one of the highest rates of gender-based violence in the world due to several socio-cultural norms (poverty, unemployment, low women’s empowerment, etc.) that are deeply rooted to Apartheid [59]. COVID-19 confinement reinforced existing abuses of South African women and increased their risk of HIV infection. Studies have demonstrated that South African women with violent partners are more likely to acquire HIV compared to those who did not experience partner violence [60,61]. Women, especially those living in poverty, cannot easily flee or fight back as they are financially and socially dependent on their male partners. Many have been psychologically traumatized by the domestic abuse that they have experienced.

High rates of HIV and TB in South Africa are consistent with income inequality, which worsened during the COVID-19 lockdown. In the report “Men, Women and Children: Findings of Living Conditions Survey 2014/2015” (2018), about one in two adults were already under the upper-bound poverty line before the arrival of the pandemic [61]. Even during its most flourishing economic era, South Africa experienced high levels of unemployment. Today, approximately 25 million out of 40 million people in the working-age population are jobless and without legal income [62]. The COVID-19 pandemic has further expanded the pool of people living in poverty, with low-wage workers suffering four-times more job losses than high-wage workers [62]. Inactivity and self-isolation are associated with high-risk activities for contracting HIV, such as having transactional sex in exchange for money, food, or drugs [63], committing crimes [64], and delaying to access healthcare facilities [65].

Housing structures in South Africa, especially in underdeveloped segregated urban areas, are important vectors for COVID-19, TB, and other airborne disease transmission. During confinement and cold winter weather, people crowd into small, poorly ventilated spaces which contribute to COVID-19 and TB disease propagation. Thus, while many urban areas may benefit from lockdowns to prevent virus transmission, conditions in overpopulated urban townships in South Africa likely experience the opposite effect. Models from the Stop TB Partnership report predict an excess of 6.3 million TB cases and 1.4 million TB deaths globally by 2025, exacerbated by a 3-month lockdown and 10 month economic restoration, with South Africa predicted to be one of the most affected countries [66].

A recent study indicates profound disruptions in TB prevention and control due to SARS-CoV-2 measures [67]. COVID-19 is likely to impede TB treatment and diagnostic services. Reasons for this are suggested to be: (1) diversion of resources that are now emphasized to manage the COVID-19 pandemic; (2) health service, politics, and media focus on the pandemic management and responses; (3) poor quality of healthcare due to stress and anxiety factors among the healthcare personal, self-isolation, and infection; and (4) fear of COVID-19 infection at healthcare facilities, which discourages visiting TB services [67]. Furthermore, testing and strict enforcement of infection control protocols and separation of people who have either HIV concurrent with TB or SARS-CoV-2 might not be feasible in hospitals due to overcrowding [68].

#### 3.2.2. Limited Access to Healthcare Resources

COVID-19 has severely disrupted South Africa’s healthcare system. Facilities and resources were reallocated to treat COVID-19 patients, leaving other patients with major diseases, like HIV or TB, at risk of not being treated or of developing complications. The arrival of COVID-19 led to a 25% reduction in TB detection worldwide and a potential increase of 13% in TB mortality [69]. With an influx of COVID-19 patients admitted to medical facilities, South Africa’s population feared risking infection at hospitals and clinics, and thus limited seeking services. Consequently, a reduction by half was observed in the number of TB tests performed and in the collection of HIV and TB medication in different provinces in South Africa [70,71]. In addition, the Global Fund to fight AIDS, TB and malaria (Global Fund) reported that more than 62% of health facilities in Africa did not have enough personal protective equipment (PPE) items for health workers (including masks, disinfectants, gloves, and hand sanitizers), which further burdened the ability of the healthcare system to safely treat COVID-19, HIV, and TB patients [72]. These consequences directly impeded efforts made by South Africa to reduce incidence rates of HIV and TB over the last few years.

HIV and TB programs in South Africa have experienced complications in meeting their projected targets. In a 2019 report on the “Mid-term review of the national strategic plan for HIV, TB and STIs 2017–2022”, South Africa was already underperforming on several aspects regarding HIV and TB [73]. For example, the HIV incidence rate decreased in 2019 but at a slower rate than in previous years, which makes it difficult for the country to reduce incidence by 50% by 2022 [73]. Similarly for TB, the country experienced an increase in TB death rate and the number of patients lost to follow-up in 2019 [73]. Equally concerning, is that the report shows that local municipalities are not prioritizing HIV and TB funding in their budget processes. In 2021, the South African government decided to extend its plan for HIV and TB to 2023 (instead of 2022 as initially planned), to recover from the damages brought by COVID-19 [74]. The reduction of the national health budget by 250 million dollars in early 2020 stifled efforts to effectively combat COVID-19 while still supporting other infectious disease programs. Other programs, like the Global Fund, have provided emergency funds for low-income countries to fight against COVID-19 and help them maintain their HIV, TB, and malaria national targets.

Limited access to healthcare services during COVID-19, especially intensive care units (ICUs), may exacerbate stigmas experienced by HIV and TB patients. Prior to COVID-19, a review from Gopalan and Vaconcellos (2019) showed that specific groups of sick individuals, typically those with HIV comorbidities, were refused admittance to hospitals [75]. These cases were observed in a public hospital in the KwaZulu Natal Province and was justified by claims that patients were ‘too sick’ for admittance. This example demonstrates the widespread discrimination of HIV patients in healthcare settings. Patient triage guidelines, such as those developed by the Critical Care Society of Southern Africa (CCSSA), are often misused or disregarded when admitting patients to ICUs. While priorities are focused on saving patients with COVID-19 complications, it is unethical and against the South African constitution to deny access to patients with HIV or TB conditions that are life threatening. To avoid further unfair patient selection, guidelines and scoring systems must be applied by all members of the healthcare system.

#### 3.2.3. Predicted Risks for Immunocompromised and Coinfected Patients

Immunocompromised patients are at increased risk of contracting and developing a severe form of COVID-19. People living with HIV (PLWH) face increased risk for developing severe COVID-19 because the virus causes a deficit in CD4^+^ and CD8^+^ T-cells responsible for stimulating the immune system. Many initial case series and cohort studies, especially in the United States and Europe, found no clear evidence of increased risks of PWLH. More recently though, a study conducted in Western Cape (South Africa) showed that HIV was associated with a doubling of COVID-19 mortality risk (Davies, 2020) [76]. While the study may have overestimated the actual HIV-associated risk due to confounding, increased risk of mortality among PLWH patients have been observed in many other countries. A WHO report was compiled on clinical data on PLWH from 24 countries and found that the risk of developing a severe or fatal form of COVID-19 was 30% higher than patients without HIV [77]. Other studies observed higher odds of COVID-19 disease progression, hospitalization, and death with PLWH [78,79].

South Africa HIV control is hampered with coinfection with TB and other infectious diseases (hepatitis B, hepatitis C, malaria, etc.). HIV and TB infected persons are speculated to have an increased vulnerability to COVID-19 infection. A study showed that coinfected patients with HIV and TB faced an increased expression of angiotensin-converting enzyme 2 (ACE2), an enzyme attached to several membranes of our body and which also serves as a gateway for the entry of the SARS-CoV-2 virus [80]. However, the response of HIV and TB coinfected patients to COVID-19 is not yet known. Further research will clarify these associations.

#### 3.2.4. Potential Positive Outcomes of the COVID-19 Pandemic on the Fight against HIV and TB

While the implications of COVID-19 have undoubtedly had wide-reaching negative impacts on South Africa’s health systems and citizens, there remains potential for the country to capitalize on changes that have occurred since the beginning of the pandemic to improve the current situation regarding TB and HIV. There have been several advents in the form of improved digital health, contact tracing, remote care, public health reporting, and public interest/education on infectious diseases.

**Figure 3:**
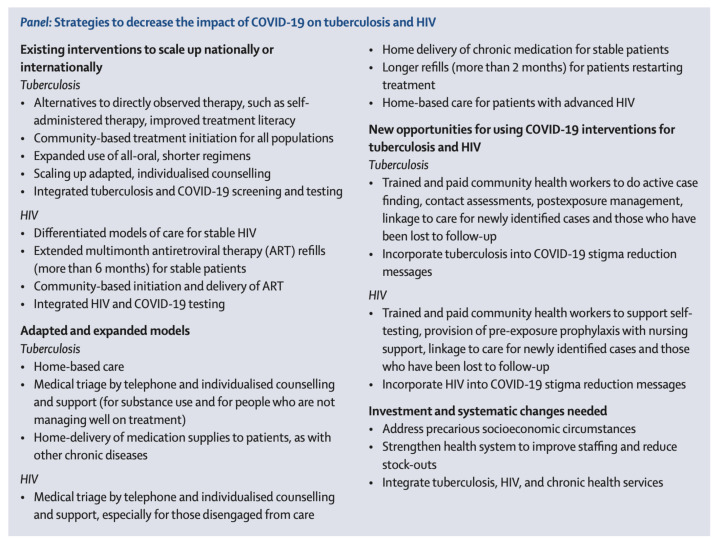
Strategies to decrease the impact of COVID-19 on tuberculosis and HIV in South Africa (Source: [81]).

The usability of digital health technologies, decentralizing TB treatment to community health workers, and supporting private health sectors and academic research institutions to provide TB testing and treatment may enhance TB treatment during a pandemic like COVID-19 [67]. Claire Keene and other researchers claim that COVID-19 measurements and actions bear potential positive effects on HIV and TB treatment services like TB screening, HIV self-testing, and the detection of patients lost to follow-up (Figure 3) [30,81].

Keene et al. (2020) maintain that COVID-19 is an opportunity for a positive long-term systematic enhancement in the healthcare system [81].

Contact tracing has previously been used in various forms to track TB cases in South Africa. A more robust centralized contact tracing system was built during the COVID-19 pandemic [82]. The system relied on door-to-door testing and tracing carried out by community health workers; however, as the pandemic wore on, the system grew more robust and reliable [82]. The similarities of TB and COVID-19 transmission lend to the potential for expertise gained in COVID-19 contact tracing to be applied for use with TB [83]. The further development of this framework and potential transition into a decentralized system could be a major benefit to the South African public health system [83,84].

Furthermore, COVID-19 necessitated tele-health and remote management of diseases like TB and HIV. This has allowed for the expansion of these services in South Africa, especially for mild and moderate cases [85]. Public health reporting, health literacy, and infectious disease awareness have all increased due to COVID-19. This provides an opportunity for funding to be leveraged by the South African government to improve the public health systems responsible for combatting HIV and TB.

## 4. Discussion

### 4.1. Insights from Reviews

This case study can be considered a high-level review of existing research linking the concomitant effects of COVID-19 with the HIV and TB epidemics in South Africa. Although non-exhaustive, its content illustrates the impactful changes that the COVID-19 pandemic have catalyzed on this landscape.

South Africa’s public health challenges are particularly nuanced. A multiplicity of systemic issues placed South Africa in a particularly tenuous position from the start of the COVID-19 pandemic. Although the government’s initial response was met with praise, the already strained health system became swiftly and repeatedly overburdened. This was exacerbated by social strife which swept across the country at various points throughout the pandemic. Underserved populations suffer the worst case numbers, fatality rates, and social and financial hardships due to the pandemic. These same populations already suffer from higher rates of HIV and TB. Unequal disease burden from COVID-19 correlates closely with existing socioeconomic strata, and inequality gaps are further widened as the country deals with sweeping economic decline. Those living in poverty tend to have little to no buffer to cope with unexpected catastrophe. Many poor families have experienced a loss or reduction of their income and job loss. With restrictive lockdowns also came an increase in violence against women.

While the country has significant prior experience with handling infectious disease outbreaks, social stigma around infectious disease, distrust in government services, and ineptitude of the public health system has severely disadvantaged at-risk communities. Access to health services was limited and, in some cases, fully restricted for HIV- and TB-positive individuals. Furthermore, testing for these diseases was hampered by lockdowns and decreased mobility. This left many individuals potentially unable to receive testing during a period where at-risk behavior was heightened [84]. While the understanding of the relationship between COVID-19 and HIV/TB is still being researched, it is fair to assume that immunocompromised individuals are at higher risk for developing severe cases of COVID-19, and growing evidence supports this claim [86]. As such, the long-term implications of the disruption of South Africa’s healthcare system during the first year of the pandemic have yet to be realized.

Socioeconomic status remains the most important indicator of burden from HIV, TB, and now COVID-19. The economic contraction attributed to COVID-19 restrictions in 2020 can be seen as a devastating blow to an already dire situation in the country. With unemployment rates now topping 34%, South Africa now holds the highest unemployment rate in the world [87]. The consequences from financial catastrophe of this magnitude are devastating and are weakening an already overburdened public health system. As HIV and TB are both resource-intensive epidemics, the capacity to combat these illnesses on a country level may be permanently hampered as evidenced by the government’s decision to delay the creation of a new national strategy for HIV/TB until 2024 [74].

Advancing equitable health access is crucial in the campaigns against HIV and TB. As seen in this literature review, factors like digital technologies and diagnostics play a pivotal role in identifying individuals that need immediate care and predicting future disease outbreaks. However, these tools must be accessible to all, particularly to women to bridge the gender divide, and more generally to underprivileged and rural communities to have a consequent impact on HIV and TB control within the South African population. This means the South African government must avoid overspending on infrastructure and development within metropolitan areas. Rather, it faces an increased need to bolster investment on novel health technologies and programs that promote health education. These same novel technologies must also be applicable and deployed for use in underprivileged communities.

The need to reduce stigma and promote human rights for people living with HIV/TB has increased in importance since the arrival of COVID-19. In this regard, initiatives responsible for the protection of these marginalized groups have led to stronger initiatives around availability, accessibility, acceptability, and quality of services [83]. Further support will be needed for these community-driven programs to lead effective campaigns and widen their reach.

A growing cloud of doubt is shading South Africa’s path forward for infectious disease control. The ongoing COVID-19 pandemic has generated profound effects on the progress that the country has gained thus far on HIV and TB. These effects will surely remain present for years to come. Despite this, it is possible that lessons learned from the COVID-19 pandemic will eventually contribute to public health progress. New frameworks and technologies developed to combat COVID-19 may eventually be integrated as assets in the campaigns against HIV and TB.

The shortcomings of the current healthcare system are widely recognized, and for nearly two decades the South Africa government has been engaged in an ambitious struggle to enact universal health care (UHC) with the goal of ensuring “that everyone has access to appropriate, efficient, affordable and quality health services” [88]. They envision tackling the current inequities and attaining UHC by implementing National Health Insurance (NHI), a financing mechanism that would allow everyone to take advantage of both public and private services [89].

### 4.2. Limitations

This case study is accompanied with some important limitations. Firstly, the COVID-19 situation in South Africa and globally is dynamic and therefore the details of this report are limited to the time of publication and the information available at this time, namely December 2021. Time will tell if the statements expressed will hold true as the pandemic wears on. Secondly, the materials and literature reviewed in this case study are by no means exhaustive and should not be treated as such. As we have only reviewed papers and reports written or translated in English, information and insights in other languages were not evaluated or discussed. This is an important limitation, as articles and reports in local languages are therefore excluded from our analysis. Utilization of non-English language sources in future reviews would ensure generalizability and reduce the risk of bias in the types of outcomes that are presented. Lastly, research on these topics is novel and the correlation of these issues is in the early phases of research. Further research is required to investigate the correlation of disease processes between COVID-19 and HIV/TB, as well as epidemiological correlations between these diseases.

### 4.3. Discovery of the SARS-CoV-2 B.1.1.529 Variant (Omicron)

As this case report is being written, a new variant of the SARS-CoV-2 virus, B.1.1.529 (Omicron), has been discovered in South Africa and is rapidly spreading. It is vital to note that, although the variant was first sequenced in South Africa, its origins are still unknown. The first few reported cases of the infection were among young individuals, specifically, university students [90]. The variant, discovered by the Network for Genomics Surveillance, has been succeeded by a rapid increase of COVID-19 cases in South Africa, particularly in Gauteng Province since its discovery on 24 November 2021 [91]. The variant has been classified as a variant of concern by the WHO [92]. As of November 2021, preliminary data indicate increasing rates of hospitalization with the Omicron variant, although this may be attributed to the increasing number of new infections associated with the variant [90]. In addition, the risk of reinfection with COVID-19 may be higher with the arrival of the new variant [90]. It is believed that COVID-19 vaccines and treatments, such as corticosteroids and IL6 receptor blockers, will remain effective in the management of severe cases of COVID-19 [90]. At this time, it is too early to state how the Omicron variant will differ in its impact on HIV and TB patients.

## 5. Conclusions

Across the world, the COVID-19 pandemic has pervasively impacted, directly and indirectly, not only the health systems of each country but many social determinants of health. South Africa bears the highest HIV burden in the world, and in this case study we have attempted to highlight some of the pandemic’s impacts and policy responses regarding the co-epidemics of HIV and TB. It is not always possible to fully predict real-world ramifications of policy decisions, especially when difficult choices must be made in a state of emergency, but it is important to critically assess their impacts and take stock. While we are far from the end of the pandemic, there are already many lessons that can be applied going forward and in preparation for the next pandemic. The lasting impacts of COVID-19 on systems and individuals in South Africa are yet to be fully realized, but we have seen significant challenges in healthcare access and a widening of pre-existing social and economic divides. As the pandemic rages on, the impacts of COVID-19 on the landscapes of the HIV and TB epidemics will inevitably become clearer. It is hoped that South Africa will emerge from this pandemic with a clearer idea to address its systemic inequities and a path toward effective control of HIV and TB within its borders.

## Data Availability

Data sharing in this case report is not applicable as no new data were created or analyzed. Figure 1 and Figure 2 were built using R [93], and the underlying data are publicly available in the references.
